# Refinement and Validation of a New Patient‐Reported Experience Measure for Hearing Loss (My Hearing PREM)

**DOI:** 10.1111/hex.70225

**Published:** 2025-03-15

**Authors:** Sian K. Smith, Helen Pryce, Georgina Burns O'Connell, Rebecca C. Knibb, Rosemary Greenwood

**Affiliations:** ^1^ School of Optometry, College of Life and Health Sciences Aston University Birmingham UK; ^2^ School of Psychology Aston University Birmingham UK; ^3^ University Hospitals Bristol and Weston NHS Foundation Trust Bristol UK; ^4^ Department of Health Sciences University of York York UK

**Keywords:** audiology, hearing loss, patient‐reported experience measure (PREM), psychometric analysis, questionnaire validation, Rasch analysis, self‐report measure

## Abstract

**Context:**

Patient‐reported experience measures (PREMs) generate insights into daily challenges experienced when living with a chronic condition and experiences of care. There are no validated PREMs to measure the experience of hearing loss.

**Objective:**

The aim of this study was to evaluate the psychometric properties of a newly developed tool, ‘My Hearing PREM’, designed to assess the experience of living with hearing loss and receiving audiology care.

**Setting and Participants:**

Adults with hearing loss (*n* = 401) were recruited from audiology clinics in Scotland and England, and non‐clinical routes such as lip‐reading classes, clinical research networks, national charity links and social media.

**Design:**

Participants completed a 27‐item PREM alongside validated scales to measure communication difficulties, loneliness, quality of life, decisional conflict and health literacy. Modern (Rasch) and traditional psychometric analysis techniques (internal consistency and construct validity) were used to assess the psychometric properties of the My Hearing PREM.

**Results:**

Factor analysis of the initial 27 items produced 3 subscales: Emotional Burden, Support and Communication, after 4 items were removed due to poor fit. Rasch analysis was carried out on each of these subscales and a further 7 items with poor fit to the Rasch model were removed. This resulted in a long‐form 16‐item (My Hearing PREM‐16) demonstrating good internal reliability (Cronbach's α = 0.91). Each subscale showed good internal reliability (0.91, 0.85 and 0.71). A short‐form (My Hearing PREM‐9) version was developed for use in clinical practice (α = 0.79). Both forms of the PREM demonstrated medium to strong significant correlations with the validated measures.

**Conclusion:**

Both the My Hearing PREM‐16 and My Hearing PREM‐9 are reliable measures with good construct validity. They provide a way for healthcare professionals to understand how hearing loss is affecting an individual's emotional well‐being, social interactions and communication. Ongoing research is exploring the feasibility of My Hearing PREM in routine audiology practice.

**Patient or Public Contribution:**

We developed the project in collaboration with members of the public who have lived experience of hearing loss, recruited through Aston University and volunteer networks connected to audiology services. Additionally, we engaged with individuals more likely to be impacted by hearing loss, including adults with learning disabilities, older adults in residential care, and members of South Asian communities (Bangladeshi, Indian and Pakistani). These stakeholders provided valuable feedback on the study's aims, the content and format of the My Hearing PREM items, the survey design and recruitment strategies.

## 1 Introduction

Globally, hearing loss is the third most common condition, affecting around 430 million people [1, 2]. By 2050, the number of people living with hearing loss is expected to rise to 700 million worldwide [1, 2, 3]. Given the chronic nature of hearing loss, understanding what it is like to live with hearing loss from the patient's perspective is crucial [4]. The psychosocial effects of hearing loss are well‐documented and include social withdrawal, reduced mental health (such as increased anxiety and depression), and a decline in overall quality of life [5]. The rise in qualitative work in audiology exploring the lived experience of hearing loss has highlighted the emotional, communication and social difficulties faced by individuals with hearing loss which can result in feelings of frustration, loneliness, poor sense of identity and a diminished sense of independence [6, 7, 8, 9].

The experience of hearing loss requires continuous effort (work) to manage and navigate different social interactions in everyday life [10]. This work can be understood in terms of illness work (coping with and responding to hearing loss itself in everyday life) and treatment work (such as accessing audiology care and managing hearing devices) [11]. Undertaking illness and treatment work is influenced by a person's social skills (the ability to organise help from others), social capital (access to informational, material and financial resources), functional performance (possessing the cognitive capacity to complete the work) and structural resilience (the extent to which a patient's social network can help them cope through the mobilisation of psychological and social resources) [11, 12, 13]. Recognising the hidden challenges that patients face can lead to more empathetic care and tailored support [12].

In a move away from focusing on the technological aspects of audiology rehabilitation, there has been a shift towards patient‐centred care within hearing healthcare [14, 15, 16]. Within this approach, patients are treated as equal partners in decisions about their care and work in collaboration with healthcare professionals and family members/communication partners [17]. Importantly, patient‐centred approaches typically result in positive outcomes, such as enhanced patient‐healthcare professional relationships, increased patient satisfaction, better adherence and higher patient engagement [18]. The growing interest in patient‐centred care has also brought attention to the concept of ‘lifeworld’, although its application in audiology is still in its infancy [10, 19]. Lifeworld refers to a person's subjective world (emotions, views and values) and how it is shaped by cultural, social and personal meanings [20, 21]. Given the ability to hear and interpret sounds is critical for communication, social interaction and navigation within the social world, hearing loss can significantly affect how people experience their lifeworld (e.g. relationships, sense of identity) [8, 9, 19]. For example, the stigma associated with hearing loss can shape a person's lifeworld, leading to social isolation and disconnection [6, 22]. Hearing assistive technology (e.g. hearing aids, cochlear implants) can potentially alter aspects of the lifeworld experienced affected by hearing loss, such as social interactions and personal identity [23], although the residual problems of hearing loss remain and cannot be cured [24].

In an era of patient‐centred care, gaining insight into a person's lifeworld can enable healthcare professionals to deliver more personalised and supportive care [20, 25]. In clinical settings, this involves facilitating a dialogue centred on exploring the patient's lived experience [25]. However, in time‐limited consultations, it may be difficult to deeply understand how individuals perceive and manage their hearing loss. To address this, interventions are needed that allow patients to efficiently share their experiences in a meaningful but concise way. One approach to achieving this is by developing and implementing a patient‐reported experience measure (PREM) [26].

PREMs are self‐report questionnaires that are designed to learn how a person feels about living with and managing their condition and treatment. They capture a person's condition, including their emotions, feelings, challenges and perceptions of interactions with healthcare professionals and services [26, 27]. This data in turn can help to inform service changes and improvements [26].

PREMs are different from patient‐reported outcome measures (PROMs). In audiology, PROMs typically assess the functional impact of hearing loss and treatment on a person's life [28, 29, 30, 31, 32, 33, 34], as opposed to the experience itself. For example, a PROM might ask about a person's ability to concentrate in various listening situations or hear speech in background noise, whereas a PREM would explore the feelings associated with communication, such as how tiring, frustrating or exhausting it feels. Recently, PROMs have been developed and validated to assess: empowerment‐related outcomes, including self‐efficacy, participation and control outcomes associated with social behaviour and perceptions [35], such as the ability to follow a lecture or engage in group discussions [36], and perceived listening effort in daily life for the adult cochlear implant population [29].

Since PROMs and PREMs offer complementary insights—capturing functional outcomes and the affective aspects of living with a condition and patient care, respectively—it is important to measure them both concurrently in clinical practice [27, 34]. For instance, an individual may feel understood and supported when seeking help, yet be dissatisfied with the outcome, such as feeling disappointed that their hearing aids do not meet their expectations or are uncomfortable to wear. Conversely, another person might perceive their hearing aids as beneficial but feel unsupported and unheard during the process of seeking assistance. PREMs are also different from patient satisfaction measures which assess whether a person's expectations of care were met. This could include aspects such as the quality of care received, the ease of accessing services and satisfaction with the overall environment of the facility [34, 37].

While PROMs have been developed and routinely used in audiology [38], there are currently no validated PREMs to measure patient experience of hearing loss. To this end, as part of the Hearing Loss and Patient Experience (HeLP) study [39, 40], we developed My Hearing PREM—the first hearing loss‐specific PREM designed to capture the lived experience of managing and coping with hearing loss from the individual's perspective—encompassing their emotions, difficulties and perceptions of support. The goal was to create a validated measure of patient experience of hearing loss in accordance with international guidelines [41, 42, 43]. Given the lack of formal guidance for developing and evaluating PREMs psychometrically [26], we used the Consensus‐Based Standards for the Selection of Health Measurement Instruments (COSMIN) [41, 42, 43] and drew on existing PROM scale development work in audiology [35, 36, 44] to guide PREM development and validation.The process of measurement development comprised the following phases: (1) conceptualisation of the lived experience of hearing loss, (2) PREM item generation, (3) content evaluation of PREM prototype, (4) item refinement using traditional (factor analysis) and modern psychometric techniques (Rasch analysis), and assessment of the reliability and validity of the tool (test–retest reliability, construct validity, internal consistency).

The first phase involved an in‐depth qualitative investigation with key stakeholders (adults with hearing loss, carers and clinicians) to explore how hearing loss is experienced across the life course and supported by significant others [40, 45]. This qualitative work and a review synthesis led to the development of our evidence‐based conceptual model of the lived experience of hearing loss [19]. At the heart of our model is the core category of individual responsibility, which captures the sense of responsibility participants felt in various aspects of their hearing loss. This includes feeling accountable for how they hear (their auditory experience), seeking professional help and social support, understanding their options and managing hearing technology (patient‐centred care), recognising how their hearing differs from others (social comparison), and managing the emotional and cognitive effort needed to cope with daily communication challenges (agency and capability) [19]. In Phase 2, the research team (S.S., G.B.O'C., H.P.) independently created a list of potential PREM items based on the conceptual model and interview data. They met regularly to refine the items and resolve any discrepancies. The aim was to develop clear, concise items that reflected participants' experiences, using their language whenever possible [39]. Following this, Phase 3 engaged with key stakeholders (Patient and Public Involvement and Engagement [PPIE] groups, clinicians, researchers, commissioners) and used cognitive, think‐aloud interviews with adults with hearing loss to appraise the content validity (relevance, clarity, acceptability, comprehensiveness) of the PREM prototype [45, 46]. This resulted in a 27‐item PREM, accompanied by a 5‐point frequency‐based response scale (Always, Most of the time, Sometimes, Rarely, Never).

The current paper focuses on Phase 4, in which we sought to further refine and validate My Hearing PREM (27 items) using modern and traditional psychometric approaches. A quantitative survey study was conducted, and data were analysed using both exploratory factor analysis and Rasch analysis to assess the psychometric properties of My Hearing PREM and remove items of poor quality. Traditional psychometric techniques were used to assess the reliability and validity of My Hearing PREM. We also sought to develop a short form with similar psychometric properties to support routine use in busy environments.

## 2 Methods

### 2.1 Development of My Hearing PREM

#### 2.1.1 Participants and Procedure

To refine My Hearing PREM (27 items) and assess its reliability and validity, adults aged 16 years and over with hearing loss and cognitive capacity to giveinformed consent were invited to participate in the study. Potential participants were recruited from audiology clinics in the UK located in Tayside (Scotland), Bath and Bristol (England) and non‐clinical settings, such as lip‐reading classes, clinical research networks, national charity links and social media. Invitation emails were also sent to participants who participated in the qualitative interviews as part of Phase 1 described above [10, 19]. Participants were eligible to participate regardless of whether they used hearing aids or not. Based on international guidelines for both exploratory factor analysis and Rasch analysis, a minimum sample size of 300 participants was set, as recommended for optimal scale development [47].

My Hearing PREM and a series of other validation measures (described below) and demographic questions (e.g. age, gender, education, ethnicity, health literacy, hearing aid use) were administered via an online survey using Qualtrics. My Hearing PREM comprised a 5‐point Likert response scale from ‘Never’ to ‘Always’, but excluded response options, ‘I don't know’ and ‘not applicable’, to encourage reflection, prevent disengagement and ensure data completeness. These options could lead to missing data, limit the ability to analyse patterns across respondents, and reduce standardisation across responses. Additionally, the temporal response format allows participants to report irrelevant experiences as ‘never’ or ‘rarely’, ensuring that all responses remain meaningful (see Supporting Information S1 for PREM scoring details). The first page of the survey presented a summary of the HeLP project and the participant information sheet. All participants gave their consent to participate by clicking a consent box before accessing the survey questions. Participants were instructed to respond to the items based on their experiences over the last week. Participation did not involve any fees or incentives. In total, the full survey took about 20 min to complete.

Members of the research team (H.P., S.S., G.B.O'C.) approached potential participants in the waiting area of the audiology clinics and invited them to complete the online survey on the study's iPad tablet or by downloading a QR code that linked directly to the survey. If participants required assistance with completing the survey (e.g. having questions read aloud or help using the tablet), a private clinic room was available to ensure they could respond in privacy. Participants also had the option to complete a paper‐based version of the survey. They were provided with a stamped envelope that included the return address, allowing them to mail it back to the HeLP study team at Aston University, Birmingham.

Test–retest reliability assesses how consistent and stable participants' responses are when they are asked the same questions at different time points. The test–retest reliability of My Hearing PREM was assessed by asking participants to complete the survey again 2 weeks after their initial survey completion. Participants were also asked whether they had experienced any changes in their hearing or consulted a healthcare professional during this period. Consenting participants received an email with a link to the retest survey, enabling the anonymous matching of their responses with the original survey data.

Ethics approval was provided by West of Scotland Research Ethics Service (approval date: 6 May 2022 ref22/WS/0057) and the Health Research Authority and Health and Care Research Wales Approval (approval date: 14 June 2022; IRAS project ID: 308816).

#### 2.1.2 Patient and Public Involvement and Engagement Contributions

Throughout the HeLP, we have endeavoured to optimise a wide range of individuals to participate in informing our research methods. This is reported in detail elsewhere [19, 45]. PPIE activities during this phase informed our recruitment and sampling activities, checked that the survey we compiled was acceptable and could be easily self‐administered and understood. PPIE contributors tested out our electronic administration of the survey and reported any practical or interpretative issues that required fixing.

#### 2.1.3 Validation Measures

Participants completed the following measures to assess the convergent construct validity of the refined My Hearing PREM (short and long versions). These scales have been validated for a general population and have excellent reliability and validity. They were specifically chosen as previous research suggests these variables (e.g. loneliness) are associated with hearing loss [5]. In the survey, the measures were presented in the following order.

##### 2.1.3.1 Revised Quantified Denver Scale of Communication Function

The revised Quantified Denver Scale (QDS) of Communication Function consists of 5 items measuring self‐reported communication function. It is scored on a five‐point Likert scale (Strongly disagree to Strongly agree) with scores ranging from 5 to 25, with scores > 11 indicating some difficulty with communication [48]. The scale has excellent internal consistency (Cronbach's α = 0.97) [48].

##### 2.1.3.2 Decisional Conflict Scale (Low Literacy Version)

Decisional conflict (uncertainty in decision‐making) was assessed using the low literacy version of the Decisional Conflict Scale (DCS) [49, 50]. This version consists of 10 items with three response options of ‘yes’, ‘don't know’ or ‘no’. There are four subscales: informed, values, clarity and support. The ten items were summed, divided by 10 and multiplied by 25. Scores range from 0 (no decisional conflict) to 100 (extremely high decisional conflict). Thus, lower scores indicate less conflict and greater confidence in decision‐making. Higher scores indicate more conflict and uncertainty.

Cronbach's α for the total scale and subscales (values, uncertainty and informed) have been reported as good (α ≥ 0.80), except for the Support subscale, which had lower reliability (α = 0.47) [49].

##### 2.1.3.3 Health Literacy

Functional health literacy was measured using three items in three different areas: (1) problems learning health information: ‘How often do you have problems learning about your medical condition because of difficulty understanding written information?’; (2) help with reading: ‘How often do you have someone help you read hospital materials?’ and (3) confidence with filling in forms: ‘How confident are you filling out medical forms by yourself?’ [51]. All items use a 5‐point Likert scale – for items 1 and 2, the response options are ‘never’, ‘occasionally’, ‘sometimes’, ‘often’, and ‘always’, and for item 3, the options are ‘extremely, ‘a lot’, ‘somewhat’, ‘a little’ and ‘not at all’. Responses are scored on a Likert scale from 1 to 5. After reverse‐scoring the item addressing confidence with forms, responses to the three items were summed (scores ranged between 3 and 15), with higher scores indicating a higher level of health literacy. Given the highly skewed distribution of scores, we decided to categorise them into two groups: inadequate health literacy (score of 11 or below) and adequate health literacy (score of 12 or above). This cut‐off point aligns with previous studies that have used this tool where similar proportions of inadequate health literacy were observed [52, 53, 54, 55]. This measure has good internal reliability (α = 0.76) [55].

##### 2.1.3.4 EQ‐5D‐5L

This is a five‐item standardised measure of health‐related quality of life [56, 57]. It is commonly used in clinical and economic health assessments to provide a simple, generic measure of health‐related quality of life for clinical and economic appraisal. It consists of five dimensions, each reflecting different aspects of health: mobility, self‐care, usual activities, pain/discomfort and anxiety/depression. Each dimension has five levels of severity from ‘No problems’ to ‘Extreme problems’. In addition, the EQ‐5D‐5L includes a visual analogue system (VAS) where respondents rate their overall health on a scale from 0 to 100, with 0 representing the worst imaginable health and 100 representing the best imaginable health. We have used the formula for scoring the measure from Devlin (2018) which used a time trade‐off and discrete choice experiment to generate values for the 3125 EQ‐5D‐5L health states [57]. The EQ‐5D‐5L has demonstrated good reliability (α = 0.79) [58].

##### 2.1.3.5 UCLA Loneliness Scale

This is a brief, validated, three‐item scale measuring perceived social isolation [59]. Respondents are asked to indicate how often they feel that they: lack companionship, feel left out and feel isolated from others. It is scored on a 3‐point Likert response scale (Hardly ever, Some of the time, Often). Total scores are calculated by adding scores on three items, with scores ranging from 3 to 9 and higher scores indicating greater levels of loneliness. This measure has been shown to have acceptable internal reliability (α = 0.72) [59].

##### 2.1.3.6 ICEpop CAPability Measure for Adults (ICECAP‐A)

The ICECAP‐A measures quality of life and well‐being in terms of the following five capabilities: stability (an ability to feel settled and secure), attachment (an ability to have love, friendship and support), autonomy (an ability to be independent), achievement (an ability to achieve and progress in life) and enjoyment (an ability to experience enjoyment and pleasure) [60]. Four levels are available for each of the five capabilities, ranging from 1 not being able to experience a capability at all to 4 being able to fully experience a capability. The ICECAP‐A attempts to capture the extent to which one experiences the freedom to be or carry out what one wishes. ICECAP‐A scores were transformed into capability values using the methods detailed by Flynn et al. [61].

##### 2.1.3.7 Hearing Information

The following information regarding hearing was collected: the presence of hearing loss in one or both ears, the use of hearing aids and other hearing assistive devices such as cochlear implants, the onset of hearing loss (whether since birth, during childhood or in adulthood), and whether they were experiencing tinnitus.

##### 2.1.3.8 Demographics

Additionally, demographic information was gathered, including age, gender, gender identity, ethnicity, household size, first four postcode digits, highest educational qualification, and a description of activities over the past 7 days (e.g. working, studying).

##### 2.1.3.9 Index of Multiple Deprivation

Participants were asked for the first four digits of their postcode. The truncated postcodes were matched to the data from the indices of multiple deprivation (IMD) in England, Wales and Scotland [62, 63, 64]. IMDs are calculated based on various indicators, such as income, employment, education, health, crime and housing. In each case, the mean decile of deprivation was used averaging all those areas which shared the same first half of the postcode. For those people who lived in an area which shared the postcode across two principalities, the English index of deprivation was used.

### 2.2 Data Analysis

Data analyses were performed using IBM SPSS Statistics version 29 and the Rasch Unidimensional Measurement Models software programme [65].

The data were examined for floor and ceiling effects to ensure that no items had excessively high or low scores, which would indicate a lack of discrimination across participants. Exploratory factor analysis (Principal Components Analysis) was then performed to eliminate items that weakened internal structural validity and to identify potential underlying clusters of variables, which could suggest the presence of subscales.

Rasch analysis was performed on each subscale separately, in line with recommended guidance [66]. It can help determine person severity (respondent ability), which on our PREM represents how much of a burden hearing loss has on a person's daily life, and item severity which reflects how burdensome or challenging a particular item is. Therefore, it is estimated that respondents with a greater burden (or poorer experience) will agree more frequently with items that reflect the severe impacts of hearing loss. Further details about the application of Rasch analysis in audiology scale development can be found here [35, 36].

#### 2.2.1 Internal Consistency

After the removal of items with a poor fit, internal consistency of the remaining items was carried out. Internal consistency refers to the degree to which a set of items on a scale measure the same concept or construct. Cronbach's alpha was used to assess the internal reliability of the overall scale and subscales [67]. Typically, a Cronbach's alpha of 0.70 or higher is considered acceptable for a PREM and its subscales [68].

#### 2.2.2 Test–Retest Reliability

To assess test–retest reliability, we calculated the intraclass correlation coefficient (ICC) on the total mean score of My Hearing PREM. The ICC indicates how strongly two sets of scores are related. A high ICC score (close to 1.0) indicates strong test–retest reliability, meaning the instrument generates consistent results over time [69]. To further assess reliability, we reported Pearson correlation coefficients.

#### 2.2.3 Construct Validity

Construct validity refers to the extent to which a measure accurately assesses the specific concept or construct it is intended to evaluate. This involves demonstrating that the PREM truly reflects the lived experience of hearing loss and is not influenced by unrelated factors. Pearson bivariate correlations were used to examine correlations between the total mean score and mean subscale scores on My Hearing PREM and other validated measures. It was predicted that My Hearing PREM would have moderate significant correlations with UCLA Loneliness, QDS, DCS and Health Literacy. Correlations were categorised as large for values above 0.5, medium for values between 0.3 and 0.49, and small for values of 0.29 or lower. All tests were two‐tailed with a significance threshold set at *p* < 0.05.

### 2.3 Creating a Short Version of My Hearing PREM

To provide clinicians and service users with a shorter option, the three subscales of the PREM were added together to produce an overall score, and items were hand‐chosen in a forward stepwise manner using linear regression to demonstrate 95% of the variation of the original longer score had been described with a smaller set of data. The aim is to produce a score with fewer items.

## 3 Results

### 3.1 Sample

A total of 485 respondents clicked on the link for the survey. Of the 485 respondents, 429 consented to participate, but 27 did not begin the survey (answer at least one item), leaving 401 participants included in the analysis (Table 1). Twenty‐eight participants (7.3%) completed the survey with support from a carer (e.g. family member) or one of the researchers.

**Table 1:** Demographic information for sample (*n* = 401).^a^

Characteristic	*N* (%)
Mean age years (range)	65.30 (16–94 years) 16.03 (SD)
Age group	
16–29 years	9 (2.24)
30–49 years	56 (13.97)
50–79 years	239 (59.60)
80 years – end of life	73 (18.20)
Gender	
Female	254 (63.34)
Male	127 (31.67)
Prefer not to say	2 (0.50)
Country of residence	
England	370 (92.27)
Northern Ireland	1 (0.25)
Scotland	19 (4.74)
Wales	11 (2.74)
Mean decile of deprivation^b^ [interquartile range] (missing data, *n* = 59)	6.26 [4.87, 7.50]
Ethnic group	
White	370 (92.27)
Mixed or multiple ethnic	3 (0.75)
Asian or Asian British	5 (1.25)
Black, Caribbean or African Caribbean	2 (0.50)
Prefer not to say	3 (0.75)
Household size	
Lives alone	87 (21.70)
2 residents	178 (44.39)
3 residents	69 (17.21)
4 or more residents	48 (11.97)
Highest educational qualification	
No qualifications	17 (4.24)
School level (GCSEs)	65 (16.21)
College level (A levels, Highers)	92 (22.94)
University level	203 (50.62)
Prefer not to say	6 (1.50)
Description of activities over the past seven days	
Retired	208 (51.87)
Working	116 (28.93)
Studying	5 (1.25)
Unemployed/job seeking	3 (0.75)
Looking after home or family	13 (3.24)
Long‐term sick or disabled	22 (5.49)
Other (e.g. holiday, volunteer work)	12 (2.99)
Prefer not to say	4 (1.0)
Presence of hearing loss	
One	46 (11.47)
Both	336 (83.79)
Prefer not to say	1 (0.25)
Health literacy	
Adequate health literacy	332 (85.3)
Limited health literacy	57 (14.7)
Hearing aid (HA) device use	
Uses HA	323 (80.55)
Prescribed HA, but does not use	24 (5.99)
No	33 (8.23)
Prefer not to say	3 (0.75)
Any other devices for hearing	
Yes	45 (11.22)
No	335 (83.54)
Prefer not to say	3 (0.75)
Devices c	
Bone‐anchored hearing aid (BAHA)	4 (1.0)
CROS/BICROS aids	2 (0.50)
Cochlear implant(s)	15 (3.74)
Hearing dog	1 (0.25)
Roger devices	6 (1.50)
Other assistive devices (e.g. multi‐mic)	7 (1.75)
Awaiting cochlear implant	4 (1.0)
Comments on past surgery	3 (0.75)
Soundbridge implant	1 (0.25)
Onset of hearing loss	
Since birth	24 (5.99)
Childhood	45 (11.22)
Adolescence	11 (2.74)
Adulthood	287 (71.57)
Unsure	15 (3.74)
Prefer not to say	1 (0.25)
Tinnitus	
Yes	230 (57.36)
No	150 (37.41)
Prefer not to say	3 (0.75)
Completed survey independently	
Yes	350 (87.28)
No	28 (2.0)
Prefer not to say	3 (0.75)
Who helped complete survey	
Researcher	21 (5.24)
Partner	3 (0.75)
Researcher and carer	2 (0.50)

^a^ Figures represent mean (SD) or number (%). Where totals do not equal 100%, data are missing; where totals are more than 100%, participants could select more than 1 option.

^b^ Higher IMD scores/deciles represent ‘less deprived areas’. Deciles range from 1 to 10, where each decile corresponds to 10% of the ranked data. The lower the decile number, the more deprived the area; higher decile numbers correspond to areas that are less deprived; 1 = the most deprived 10% of areas; 10 = the least deprived 10% of areas. For the current study, 50 participants chose not to share their postcode.

^c^ One participant mentioned 2 hearing devices – Bone‐anchored hearing aid (BAHA) and assistive listening devices.

A total of 199 respondents started the retest survey, with 198 consenting to participate (a response rate of 49.4% out of the total of 401 participants). Among the 198 respondents, 8 were excluded from the analyses because they did not complete any of the PREM measure, leaving 190 participants included in the test–retest analyses.

### 3.2 Item Refinement

#### 3.2.1 Internal Structural Validity of My Hearing PREM

Principle components analysis with a varimax rotation, extracting factors (components) with eigenvalues over 1, was initially run to explore the internal structural validity of the scale (see Supporting Information S2 for details of the initial factor solution). In the first Iteration 3 items did not load onto any factor and were removed from the analysis, which was run again. In the second iteration, one item did not load and was removed. The third iteration produced a 3‐factor solution, which explained 53.26% of the variance in the data. The KMO was 0.92 indicating an adequate sample size and Bartlett's test of sphericity was 4359.432, *p* < 0.001, indicating items were not independent of each other. The determinant was 0.00001459, showing that singularity was not an issue with the data. Ten items loaded onto the first factor, which was named Emotional Burden. Six items loaded onto factor two, named Communication. Two of these also loaded highly on factor 1, but they made more conceptual sense to place them on factor two. Seven items loaded onto factor three, named Support. One of these items loaded equally well on factor 1, but the item was a better fit with factor 3 (Table 2).

**Table 2:** Factor loadings for a principle components analysis with varimax rotation for a 3‐component solution for items on My Hearing PREM.

PREM item	PREM question	Factor 1	Factor 2	Factor 3
**Emotional Burden**				
4	I feel lonely when I can't hear and others can	0.76		
6	There are certain situations where I feel really left out	0.75		
7	I avoid activities I used to enjoy because of my hearing	0.72		
3	I am frustrated by my hearing problems	0.70		
9	I worry what people think of me because I can't hear everything	0.69		
8	I have to concentrate more because of struggling to hear	0.66		
12	I have worried my hearing will get worse in the future	0.60		
10	I am confident talking to people in background noise	0.56		
13	I have felt unsafe when I am out and about because I don't hear what is going on around me	0.54		
11	I notice the problems caused by not hearing	0.50		
**Communication**				
17	I have thought about getting devices to help me hear alarms, the phone or TV		0.74	
18	It is difficult to communicate with organisations, GP surgeries, etc.		0.65	
19	Phone calls are hard for me because of my hearing		0.63	
16	I have thought about whether I need support with my hearing such as lip‐reading classes, groups or hearing therapy		0.58	
14	Trying to hear can be exhausting	0.67	0.46	
2	It is an ongoing struggle to hear others	0.61	0.40	
**Support**				
23	I am confident that professionals will listen to my point of view			0.74
22	I feel confident communicating my needs and concerns about my hearing to medical/healthcare professionals			0.73
21	Medical/healthcare professionals support me with my hearing			0.68
26	Healthcare professionals/audiologists clearly explain what to expect from audiology tests and results			0.65
15	My family and friends support me with my hearing			0.56
25	I understand what hearing testing involves			0.53
1	I feel confident telling people when I haven't heard them	0.48		0.42
	**Eigenvalues**	6.19	3.13	2.93
	**Variance explained (%)**	26.90	13.61	12.75

#### 3.2.2 Rasch Analysis

My Hearing PREM has polytomous data which makes it appropriate for Rasch analysis. All items on the PREM have the same scale rating structure for responses (a 5‐point Likert response scale from ‘Never’ to ‘Always’) meaning the threshold parameters were the same distance between them for all items. There was no missing data for the Rasch analysis because each item had to be completed to finish the My Hearing PREM, so only fully completed responses were included in the data analysis. The parameterisations of the Rasch model used for this study was the Unrestricted or Partial‐Credit Model which is the standard setting when using RUMM2030. The Partial‐Credit Model assumes that for each item there are varying distances between thresholds and there are differing amounts of response categories [65].

##### 3.2.2.1 Iterative Process of Item Reduction

The psychometric properties of each subscale were inspected using Rasch analysis and those items which showed poor properties were removed from the set of items included in each subscale and an iterative process of analysis was conducted (Table 3).

**Table 3:** Record of PREM items included or removed during Rasch analysis.

Item	Item content	Status: Included or reason for removal
**Emotional burden**		
3	I am frustrated by my hearing problems	Included
4	I feel lonely when I can't hear and others can	Included
6	There are certain situations where I feel really left out	Included
7	I avoid activities I used to enjoy because of my hearing	Included
9	I worry what people think of me because I can't hear everything	Included
13	Over the last week, I have felt unsafe when I am out and about because I don't hear what is going on around me	Included
8	I have to concentrate more because of struggling to hear	Removed^a^
10	I am confident talking to people in background noise	Removed^b^
11	I notice the problems caused by not hearing	Removed^c^
12	Over the last week, I have worried my hearing will get worse in the future	Removed^d^
**Communication**		
14	Trying to hear can be exhausting	Included
17	I have thought about getting devices to help me hear alarms, the phone or TV	Included
18	It is difficult to communicate with organisations, GP surgeries etc because of my hearing	Included
19	Phone calls are hard for me because of my	Included
2	It is an ongoing struggle to hear others	Removed^e^
16	I have thought about whether I need support with my hearing such as lip‐reading classes, groups, or hearing therapy	Removed^d^
**Support**		
1	I feel confident telling people when I haven't heard them	Included^f^
15	My family and friends support me with my hearing	Included
21	Medical/healthcare professionals support me with my hearing	Included
23	I am confident that professionals will listen to my point of view about my hearing	Included
25	I understand what hearing testing involves	Included
26	Healthcare professionals/audiologists clearly explain what to expect from audiology tests and results	Included
22	I feel confident communicating my needs and concerns about my hearing to medical/healthcare professionals	Removed

^a^ Failed 1 test of fit and borderline on other tests (failed chi‐square and borderline positive residual fit with 2.299).

^b^ Failed 2 tests of fit (chi‐square and high positive residual).

^c^ Failed 1 test of fit and borderline on other tests.

^d^ Failed 3 tests of fit (failed fit residual, chi‐square and F‐stat).

^e^ Failed 2 tests of fit (chi‐square and F‐stat).

^f^ Items not removed due to lowering the reliability scores on PSI and Cronbach's Alpha and because of their fittingness with the latent traits conceptually in addition to their appropriateness for clinical application.

##### 3.2.2.2 Emotional Burden Subscale

###### 3.2.2.2.1 Item Fit Statistics and Reliability

Analyses were initially performed on the 10 potential items, revealing a poor fit to the Rasch model (χ^2^ = 99.29, *p* < 0.01). Four items were identified (items 8, 10, 11 and 12) with problematic psychometric properties (Table 4). Items 10 and 12 showed high positive fit residuals (4.8 and 3.2), indicating poor fit while item 11 was borderline (2.3). These items were less effective in discriminating between different levels of emotional burden in individuals with hearing loss. Additionally, items 8, 10 and 12 had significant chi‐square probabilities (*p* < 0.05), further confirming poor fit to Rasch model. As a result, these items were removed. After removing the misfitting items, the emotional burden subscale demonstrated excellent reliability, with a Cronbach's alpha of 0.87 and a Person Separation Index (PSI) of 0.86, indicating the subscale could reliably distinguish between varying levels of emotional burden.

**Table 4:** Fit statistics of PREM to the Rasch model.

Analysis and subscale name	Number of items	Item fit residual		Person fit residual		Chi‐square interaction			PSI with extremes^a^	Cronbach's alpha	Unidimensionality *t*‐tests (CI)			
		Mean	SD	Mean	SD	Value	df	*p*			Number of significant tests	Out of	%	Lower bound 95% CI
Emotional burden initial	10	0.57	2.42	−0.31	1.27	99.29	60	0.001073	0.89	0.89				
Emotional burden final	6	0.46	1.71	−0.36	1.09	47.72	42	0.251553	0.86	0.87	24	401	5.99%	0.04%
Communication initial	6	0.35	1.29	−0.27	1.04	81.2	36	0.000024	0.85	0.86				
Communication final	4	0.21	1.53	−0.33	0.97	33.69	20	0.028308	0.80	0.84	10	401	2.49%	0.004%
Support initial	7	0.10	2.00	−0.38	1.05	96.13	42	0.000004	0.75	0.77				
Support final	6	−0.08	1.13	−0.37	0.94	54.58	30	0.004002	0.69	0.70	20	395	5.06%	0.03%

^a^ The PSI with extremes reported to show the PREM's reliability across a diverse range of participants/patients.

##### 3.2.2.3 Communication Subscale

###### 3.2.2.3.1 Item Fit Statistics and Reliability

Initial analysis of the six potential items indicated poor fit to the Rasch model (χ² = 81.20, *p* < 0.01) (Table 4). Two items (2 and 16) displayed problematic fit. Item 2 failed two fit tests (chi‐square *p* < 0.05, F‐stat = 3.7), while Item 16 failed three tests (fit residual = +2.7, chi‐square *p* < 0.05, F‐stat = 3.32). Both items did not fit the model at the 5% and 1% significance levels and were removed (Table 3). After removing Items 2 and 16, the Communication subscale demonstrated excellent reliability with a Cronbach's alpha of 0.87 and a PSI of 0.80.

##### 3.2.2.4 Support Subscale

###### 3.2.2.4.1 Item Fit Statistics and Reliability

Initial analysis of seven potential items indicated poor fit to the Rasch model (χ² = 96.13, *p* < 0.01) (Table 4). Three items (Items 1, 22 and 23) showed problematic fit. Items 22 and 23 failed three fit tests (fit residual = −3.1 and −2.8, chi‐square *p* < 0.05, F‐stat = 5.3 and 5.4), while Item 1 failed two tests (chi‐square *p* < 0.05, F‐stat = 3.1). Items 22 and 23 exhibited high negative fit residuals, suggesting redundancy due to dependency between them.

Further analysis revealed local dependency between Items 22 and 23 (correlation = 0.328, above the acceptable threshold) [70]. This dependency was conceptually expected, as confidence in healthcare professionals' attentiveness could influence an individual's confidence in communicating their needs. Given their redundancy, Item 22 was removed, while Items 1 and 23 were retained for their clinical relevance. After removing Item 22, the Support subscale achieved acceptable reliability with a Cronbach's alpha of 0.70 and a PSI of 0.70.

###### 3.2.2.4.2 Internal Consistency

The final My Hearing PREM‐16 contained 16 items also had excellent internal consistency with a Cronbach's alpha of 0.91 (see Table 5).

**Table 5:** Internal consistency statistics for the PREM and its subscales.

	PREM‐16	Subscales			PREM‐9
		Emotion	Communication	Support	
Cronbach's α	0.91	0.91	0.71	0.85	0.79

##### 3.2.2.5 Test–Retest Reliability

The test–retest analyses (ICCs and Pearson's bivariate correlations) indicated that the My Hearing PREM‐16 scale has good to excellent retest, regardless of reported changes in hearing or contact with a healthcare professional (where good is classified as 0.75–0.89 and excellent is classified as > 0.90) (Table 6). The Pearson's bivariate correlation also demonstrated excellent test–retest reliability with a strong effect size.

**Table 6:** Test–retest reliabilities (intraclass correlation and Pearson bivariate correlation).

Sample^a^	Intra‐class correlation	Pearson's bivariate correlation
Whole sample (*n* = 190)	0.89 (0.85–0.92)	0.89, *p* < 0.01
No reported changes to hearing (*n* = 111)	0.89 (0.84–0.92)	0.89, *p* < 0.01
Reported changes to hearing (*n* = 19)	0.88 (0.70–0.95)	0.90, *p* < 0.01

^a^ Data missing for 60 participants.

##### 3.2.2.6 Construct Validity

Construct validity was assessed by calculating Pearson correlations between My Hearing PREM‐16 and selected measures examining related constructs (Table 7). My Hearing PREM‐16 significantly correlated with all scales. As expected, strong correlations were found for Denver's communication and loneliness meaning that higher scores on My Hearing PREM‐16 (indicating greater burden/poorer experience) were significantly associated with greater levels of loneliness and perceived communication difficulties. All other correlations were weak to moderate but significant, apart from with the reading subscale of the Health Literacy scale.

**Table 7:** Correlations (Pearsons) between PREM‐16 (and 3 subscales) and PREM‐9 and other measures to test construct validity.

Measure	PREM‐16	PREM‐16			PREM‐9
		Subscales			
	Full	Emotion	Communication	Support	
	*r*				
**QDS**	0.72*	0.69*	0.59*	0.40*	0.69*
**DCS total**	0.31*	0.21*	0.08	0.48*	0.27*
**DCS certain**	0.33*	0.24*	0.13*	0.43*	0.30*
**DCS inform**	0.18*	0.10**	‐0.03	0.38*	0.15*
**DCS values**	0.14*	0.06	‐0.03	0.34*	0.11**
**DCS support**	0.43*	0.33*	0.19	0.51*	0.39*
**Health literacy learn**	‐0.30*	‐0.26*	‐0.28*	‐0.14*	‐0.29*
**Health literacy read**	‐0.07	‐0.06	‐0.15*	0.07	‐0.08
**Health literacy confident**	‐0.17*	‐0.13*	‐0.15*	‐0.13**	‐0.15*
**EQ‐5D‐5L**	‐0.31*	‐0.30*	‐0.29*	‐0.13*	‐0.28*
**UCLA**	0.61*	0.60*	0.46*	0.34*	0.56*
**ICECAP‐A**	‐0.48*	‐0.44*	‐0.34*	‐0.36*	‐0.43*
**Mean IMD deprivation decile**	‐0.25*	‐0.23*	‐0.23*	0.12*	‐0.25*

QDS, revised quantified Denver Scale of communication function; DCS, Decisional Conflict Scale (low literacy version); EQ‐5D‐5L, EuroQol‐5 Dimensions, 5 Levels; UCLA, UCLA Loneliness Scale; ICECAP‐A, ICEpop CAPability measure for Adults

* *p* < 0.01.

** *p* < 0.05.

Similarly, there were significant positive correlations between My Hearing PREM‐16 subscales (emotion, communication and support) and most of the measures. Support subscale correlated with all measures apart from the reading subscale of the Health Literacy scale. The Emotion subscale correlated with all but the DCS values the reading subscale of the Health Literacy scale. The Communication subscale significantly correlated with certainty DCS, loneliness, communication difficulties and all subscales of the Heath Literacy Scale.

Participants with adequate health literacy reported better scores on My Hearing PREM‐16 (total mean = 2.67, SD = 0.64) compared to those with limited health literacy (mean = 3.01, SD = 0.55) (t(387) = −3.79, *p* < 0.001). Participants with adequate health literacy also scored better on the Emotion subscale (mean = 2.82, SD = 0.83) compared to those with limited health literacy (mean = 3.26, SD = 0.85) (t(387) = –3.68, *p* < 0.001). They also scored better on the Communication subscale (mean = 3.16, SD = 0.10) compared to those with limited health literacy (mean = 3.74, SD = 0.89) (t(387) = −4.12, *p* < 0.001). There was no significant difference for the Support subscale.

### 3.3 Reliability and Validity of My Hearing PREM‐9

My Hearing PREM‐9 demonstrated good internal consistency with a Cronbach's alpha of 0.79. My Hearing PREM‐9 also demonstrated weak to strong significant correlations with all validation scales apart from the Read subscale of the Health Literacy scale (Tables 5 and 7).

## 4 Discussion

The overall aim of this study was to refine and validate a PREM to measure the experience of hearing loss and audiology care. The process led to two measures: a 16‐item (My Hearing PREM‐16 with 3 distinct subscales measuring emotion, communication and support) and a 9‐item (My Hearing PREM‐9) instrument. It represents the first study of its kind to validate a hearing loss‐specific PREM and used both traditional and modern psychometric analysis.

The results showed that both PREMs (My Hearing PREM‐16 and My Hearing PREM‐9) had a good range of psychometric properties, demonstrating acceptable internal consistency and construct validity. Specifically, each PREM (and the subscales of My Hearing PREM‐16) had Cronbach's alpha levels within the required range of 0.70 and 0.95 [71]. Construct validity was assessed by correlating scores on both PREMs with constructs that are related to the experience of hearing loss or associated with having hearing loss. The direction and strength of the correlations between each PREM, the subscales and other measures were predominately as expected. Most correlations were significant, with particularly strong positive correlations observed for loneliness and communication difficulties on each PREM, indicating that individuals who experience a negative lived experience of hearing loss are more likely to report higher levels of loneliness and problems with communication. Indeed, evidence consistently shows that hearing loss is associated with loneliness, social isolation and communication difficulties [5, 72], indicating that patients may need psychological support to cope with the emotional challenges of hearing loss. There was strong agreement between the PREM scores and quality of life measured by both the ICECAP and the EQ‐5D‐5L, showing that reporting a poor experience of hearing loss is associated with reporting poor capability and health‐related quality of life. My Hearing PREM demonstrated strong test–retest reliability, suggesting that similar responses would be given by participants, if the measure was administered multiple times [71].

A key strength of this study was the integration of both modern and traditional psychometric methods to refine and validate the PREM measures. Additionally, this study adds to the limited research using contemporary techniques to develop hearing‐specific measures, such as PROMs, and represents the first PREM in the field of audiology [29, 35, 36]. The relatively large sample size (~400) and strong test–retest response rate (~50%) suggests that participants were motivated to engage with the research and found the topic relevant to their experiences. We did not record whether respondents were recruited through the clinic or other methods (such as social media or referrals from friends). However, our clinic‐based recruitment allowed us to engage and support individuals who might not have otherwise participated, including those with limited digital skills and health literacy, learning difficulties, or dementia.

Our survey responses show that the PREM can be effectively completed by a diverse group of individuals affected by hearing loss, including those who do and do not access audiology care, such as adults with learning disabilities, cognitive decline, dementia, and those for whom English is not the first language. While self‐report data has limitations, in this case, it is necessary to capture individuals' lived experiences of hearing loss, as no objective method can fully capture these subjective perceptions. The data and voluntary response rate suggest that the PREM reflects key aspects of these experiences, with no significant issues raised in the free‐text responses or cognitive testing [46]. However, one limitation is that if the PREM is used as an outcome in a randomised clinical trial, patients would need to remain blinded to their group status to avoid bias. Additionally, using the PREM alone to compare services across geographic regions could be problematic, as variations in needs or outcomes might affect the results. Future research should consider integrating multiple data sources to provide a more complete picture of service delivery and patient experiences.

Most participants were white British and residing in England, highlighting the need for further research to assess the reliability and validity of the scale across different ethnic groups and geographic regions within the UK. Despite efforts to recruit participants from diverse ethnic backgrounds, these groups were underrepresented, reflecting both the general UK population composition [73] and existing demographic trends among audiology service users [74]. To ensure the cultural validity of the PREM across different ethnicities, future research should prioritise targeted recruitment strategies to achieve a more representative sample and explore how cultural beliefs and expectations influence PREM responses. Additionally, nearly three‐quarters (73.56%) of participants had post‐secondary education (A levels in the UK), and most (85.3%) demonstrated adequate health literacy. To enhance generalisability, future studies should include individuals with lower educational attainment and health literacy levels. Although participants varied in age, the youngest and oldest groups were underrepresented, a limitation also noted during the qualitative interviews used to develop the conceptual model [19]. Targeted strategies are needed to recruit these groups in audiology research. As noted above, wedid not record whether respondents were recruited through the clinic or other methods (such as social media or referrals from friends). Future studies should consider recording recruitment sources at the point of data collection to allow for more detailed subgroup analyses.

In the Rasch analysis, the support subscale reliability scores were borderline (PSI = 0.7; α = 0.7). While these scores are appropriate for group‐level use [75], a reliability score of ≥ 0.85 is recommended for individual‐level use. The support subscale provides a steer to clinicians on the level of support their patients experience within the health system and other sources. The support items can be used as a starting point to guide in‐depth discussions, rather than as stand‐alone metrics. Additionally, the scale can reliably distinguish between group‐level patient experiences, making it valuable for informing audiology services and commissioning.

Findings supported unidimensionality within the three components (emotional burden, communication and support), meaning each subscale measures a single latent trait. Psychometric properties of these components were evaluated using both Rasch analysis and traditional factor analysis. Item removal decisions considered Rasch model fit, practical completion time, and the impact on clinical appointments. Certain advanced analyses, such as item characteristic curves (ICCs) and differential item functioning (DIF), were excluded due to low response frequencies, which reduced statistical power. Future studies with larger sample sizes should explore these analyses further. The response categories (‘never’, ‘occasionally’, ‘sometimes’, ‘often’ and ‘always’) performed well in descriptive analyses and during our think‐aloud interviews [46]. Any issues identified during development were addressed by rewording and iterative testing. Future work could include threshold ordering, which was not feasible within the current study's timeframe.

To build on this study, a mixed methods evaluation is currently underway to determine the feasibility of implementing My Hearing PREM into routine audiology practice. This evaluation will explore its potential by examining dimensions such as acceptability, adoption, appropriateness, feasibility, fidelity, implementation cost, penetration and sustainability [76]. Further multivariate analyses are underway to examine the factors (e.g. age, education, health literacy, hearing device type, onset of hearing loss) associated with PREM responses.

## 5 Conclusion

This study is the first to refine and validate a hearing loss‐specific PREM in audiology. Both the 16‐ and 9‐item versions of the My Hearing PREM showed good psychometric properties, including acceptable internal consistency and construct validity, indicating that they are suitable for use in research and clinical settings to assess patients' experiences of living with hearing loss and receiving audiology care. This study highlights the benefits of using modern psychometric analysis techniques alongside more conventional methods to generate a robust audiology PREM. Results from the scale could have direct implications for practice by helping clinicians to understand when hearing loss is affecting an individual's emotional well‐being and social interactions, and where referral or sign posting to relevant support services or groups (e.g. hearing therapy, lip‐reading classes) might be helpful. Importantly, both tools measure hearing loss experience among young people and adults (from the age of 16 years) with any type of hearing loss, including those with and without hearing aids or other devices. This means that a clinician or researcher can administer My Hearing PREM (9‐ or 16‐item) with any adult living with hearing loss. Further research is currently underway to determine the feasibility of implementing My Hearing PREM into routine audiology service practice and using it to benchmark services or commission services better. My Hearing PREM‐16 and My Hearing PREM‐9 can be freely downloaded – https://www.aston.ac.uk/research/hls/hearing-loss-and-patient-reported-experience-help [77, 78].

## Author Contributions

**Sian K. Smith:** methodology, software, data curation, investigation, validation, formal analysis, visualisation, conceptualisation, writing – original draft, writing – review and editing, project administration. **Helen Pryce:** conceptualisation, methodology, investigation, validation, formal analysis, supervision, funding acquisition, visualisation, project administration, resources, writing – review and editing. **Georgina Burns O'Connell:** methodology, software, data curation, investigation, conceptualisation, validation, formal analysis, visualisation, project administration, writing – review and editing. **Rebecca C. Knibb:** conceptualisation, methodology, software, data curation, investigation, validation, formal analysis, supervision, funding acquisition, visualisation, writing – review and editing. **Rosemary Greenwood:** conceptualisation, methodology, software, data curation, investigation, formal analysis, validation, funding acquisition, visualisation, writing – review and editing.

## 6 Ethics Statement

The study was approved by the West of Scotland Research Ethics Service (approval date: 6 May 2022, ref: 22/WS/0057) and the Health Research Authority and Health and Care Research Wales (HCRW) Approval (approval date: 14 June 2022; IRAS project ID: 308816).

## Conflicts of Interest

The authors declare no conflicts of interest.

## Supporting information

Scoring of the PREM.

Initial factor solution.

## Data Availability

The data that support the findings of this study are available on request from the corresponding author. The data are not publicly available due to privacy or ethical restrictions.
