# Supplementary material for: Refinement and Validation of a New Patient‐Reported Experience Measure for Hearing Loss (My Hearing PREM)
Source: Health Expect. 2025 Mar 15;28(2):e70225. doi: 10.1111/hex.70225 (PMC11909470; doi:10.1111/hex.70225)
Supplement: Supplementary file 1 — Scoring of the PREM. [file HEX-28-e70225-s002.docx]

**Supplementary material 1**

**Scoring of My Hearing PREM (16 and 9 item)**

For My Hearing PREM-16 and PREM-9 instruments, each item is scored on a scale from 1 to 5. The total score is calculated as the sum of scores across all 16 items for PREM-16 or 9 items for PREM-9. Higher total scores reflect a greater burden or poorer experience. Items related to communication are reverse scored. Tables S1 and S2 detail the specific items included in each subscale for PREM-16 and PREM-9. Additional information on the scoring methodology can be found here: <https://www.aston.ac.uk/research/hls/hearing-loss-and-patient-reported-experience-help>

Table S1. Items belonging to each subscale for My Hearing PREM-16

| **Subscale** | **Item number** |
| --- | --- |
| Emotion | 1-6 |
| Support | 7-10 |
| Communication | 11-16 |

Table S2. Items belong to each subscale for My Hearing PREM-9

| **Subscale** | **Item number** |
| --- | --- |
| Emotion | 1-3 |
| Support | 4-6 |
| Communication | 7-9 |
