# Supplementary material for: Refinement and Validation of a New Patient‐Reported Experience Measure for Hearing Loss (My Hearing PREM)
Source: Health Expect. 2025 Mar 15;28(2):e70225. doi: 10.1111/hex.70225 (PMC11909470; doi:10.1111/hex.70225)
Supplement: Supplementary file 2 — Initial factor solution. [file HEX-28-e70225-s001.docx]

**Supplementary material 2**

**Initial solution -** **Principle Components Analysis with Varimax rotation and Eigenvalues set to more than 1.0. Coefficients less than 0.40 not shown.**

In the initial solution there were a number of cross loadings and three of the factors only had 2 items on each. This and the scree plot suggested that a solution with 2 or 3 factors would be more comprehensible. When a two factor and three factor solution was run, items 20, 24 and 27 did not load onto any factor above 0.4. These were removed and two and three factor solutions were run again. Item 5 did not load onto any factor above 0.4 in the 2 factor solution and in the 3 factor solution the 3^rd^ factor had only 2 items loading onto it. Item 5 was removed and 2 and 3 factor solutions were run again. The 3 factor solution made most conceptual sense and described more variance in the data.

| **Rotated Component Matrix^a^** | | | | | |
| --- | --- | --- | --- | --- | --- |
|  | Component | | | | |
|  | 1 | 2 | 3 | 4 | 5 |
| 14. Trying to hear can be exhausting | .785 |  |  |  |  |
| 7. I avoid activities I used to enjoy because of my hearing | .761 |  |  |  |  |
| 8. I have to concentrate more because of struggling to hear | .759 |  |  |  |  |
| 2. It is an ongoing struggle to hear others | .743 |  |  |  |  |
| 6. There are certain situations where I feel really left out | .738 |  |  |  |  |
| 18. It is difficult to communicate with organisations, GP surgeries etc because of my hearing | .719 |  |  |  |  |
| 4. I feel lonely when I can’t hear and others can | .707 |  |  |  |  |
| 19. Phone calls are hard for me because of my hearing | .684 |  |  |  |  |
| 3. I am frustrated by my hearing problems | .682 |  |  |  |  |
| 11. I notice the problems caused by not hearing | .653 |  |  |  |  |
| 13. Over the last week, I have felt unsafe when I am out and about because I don’t hear what is going on around me | .608 |  |  |  |  |
| 10. I am confident talking to people in background noise | .603 |  |  |  |  |
| 17. I have thought about getting devices to help me hear alarms, the phone or TV | .575 |  |  | -.413 |  |
| 9. I worry what people think of me because I can’t hear everything | .569 |  |  |  |  |
| 12. Over the last week, I have worried my hearing will get worse in the future | .565 |  | .404 |  |  |
| 16. I have thought about whether I need support with my hearing such as lip-reading classes, groups, or hearing therapy | .498 |  |  |  |  |
| 22. I feel confident communicating my needs and concerns about my hearing to medical/healthcare professionals |  | .805 |  |  |  |
| 23. I am confident that professionals will listen to my point of view about my hearing |  | .801 |  |  |  |
| 21. Medical/ healthcare professionals support me with my hearing |  | .655 |  |  |  |
| 26. Healthcare professionals/audiologists clearly explain what to expect from audiology tests and results |  | .576 |  |  |  |
| 1. I feel confident telling people when I haven’t heard them |  | .468 |  |  |  |
| 27. I worry about what a hearing test might show |  |  | .836 |  |  |
| 24. I worry about having my hearing tested |  |  | .779 |  |  |
| 20. When I go to health appointments, I have family or friends who help me with my hearing |  |  |  | .753 |  |
| 15. My family and friends support me with my hearing |  | .490 |  | .537 |  |
| 25. I understand what hearing testing involves |  |  |  |  | .653 |
| 5. When I speak to people for the first time, I tell them about my hearing |  |  |  |  | -.597 |
| Extraction Method: Principal Component Analysis.  Rotation Method: Varimax with Kaiser Normalization. | | | | | |
| a. Rotation converged in 7 iterations. | | | | | |
